# *Halobacterium salinarum* NRC-1 Sustains Voltage Production in a Dual-Chambered Closed Microbial Fuel Cell

**DOI:** 10.1155/2022/3885745

**Published:** 2022-09-12

**Authors:** Rodrigo Oliveira Goncalves, Ali Salehi, Marlon Publico, Jimmy Nyende, Nalina Nadarajah, Soheil Ghoreyshi, Padmaja Shastri

**Affiliations:** ^1^Biomedical Engineering Program, Applied Biological and Environmental Science, Centennial College, 941 Progress Avenue, M1G 3T8, Scarborough, Canada; ^2^Biotechnology Program, Applied Biological and Environmental Science, Centennial College, 755 Morningside Avenue, M1C 4Z4, Scarborough, Canada

## Abstract

Sustained bioenergy production from organisms that thrive in high salinity, low oxygen, and low nutrition levels is useful in monitoring hypersaline polluted environments. Microbial fuel cell (MFC) studies utilizing single species halophiles under salt concentrations higher than 1 M and as a closed microbial system are limited. The current study aimed to establish baseline voltage, current, and power density from a dual-chambered MFC utilizing the halophile *Halobacterium salinarum* NRC-1. MFC performance was determined with two different electrode sizes (5 cm^2^ and 10 cm^2^), under oscillating and nonoscillating conditions, as well as in a stacked series. A closed dual-chamber MFC system of 100 mL capacity was devised with *Halobacterium* media (4.3 M salt concentration) as both anolyte and catholyte, with *H. salinarum* NRC-1 being the anodic organism. The MFC measured electrical output over 7, 14, 28, and 42 days. MFC output increased with 5 cm^2^ sized electrodes under nonoscillating (*p* < 0.0001) relative to oscillating conditions. However, under oscillating conditions, doubling the electrode size increased MFC output significantly (*p* = 0.01). The stacked series MFC, with an electrode size of 10 cm^2^, produced the highest power density (1.2672 mW/m^2^) over 14 days under oscillation. Our results highlight the potentiality of *H. salinarum* as a viable anodic organism to produce sustained voltage in a closed-MFC system.

## 1. Introduction

Energy produced by microbial fuel cell (MFC) has traditionally been directed towards wastewater treatment and soil bioremediation, using mixed microbial communities including archaea [1, 2]. Energy is generated through an external electron transference mechanism, which occurs through biochemical conversion of organic material by microbes [3]. A two-chambered MFC consists of an ion-exchange membrane that separates the anode from the cathode, with electroactive microbes on the anodic side producing electrons and protons via redox reactions. The electrons are captured at the anode and are transferred to the cathode side via an external circuit [4]. Differences in microbial communities can affect electron transfer mechanisms, altering MFC output [5]. *Shewanella* and *Geobacter*, belonging to the phylum Proteobacteria, have been the predominant focus of electromicrobiology [6], and studies have shown *Geobacter* to be a dominant species on the anode surface in MFCs utilizing mixed communities from soil and water [7]. More recently, *Geobacter* was shown to dominate in power generation in both nonaerated and aerated MFCs using mixed communities to remediate metal toxicity [8]. *Geobacter* and *Shewanella* are well categorized to mediate direct electron transfer to anodes via nanowires or electron-transport proteins without the need for diffusional electron mediators [9]. Although *Geobacter* and *Shewanella* display well characterized electrogenic properties [10], they cannot withstand extreme environments. Environments contaminated with industrial processes such as mining, tanneries, and oil-refining can become hypersaline [11]. Bioremediation or desalinating of wastewater in these environments can be achieved by halophiles that are best suited to grow in conditions where salt concentrations vary between 0.3 and 3.4 M [1]. High ionic strength reduces internal resistance in microbial electrical systems, and early work with *Haloferax volcanii* determined high ionic strength (2.8 M) helped improve MFC performance [12]. However, high salt concentrations become problematic in MFCs utilizing nonhalophilic electrogenic organisms. For example, a recent study using 16 S pyrosequencing showed *Geobacter* grew well in a mixed anodic culture under salt concentrations up to 0.1 M in a single chamber MFC. However, power generation declined when salt concentration increased to 0.3 M [13].

*Halobacterium salinarum*, an extreme halophile, grows at salt concentrations of 3.5–4.5 M [14] and has been well-studied for its four transmembrane archaeal rhodopsins [15, 16]. Light energy captured by these purple membrane proteins are converted into structural changes that pump protons across the cytoplasm to the extracellular side. This proton pump creates an electrochemical gradient that helps drive cellular processes within *H. salinarum* [15–17]. Archaeal rhodopsin has been the most important biomolecule extracted from *H. salinarum* for commercial use in a variety of optometric-related technologies [18] and could be of use in bioanodes. Although proteins, over whole organisms, are an attractive alternate in bioenergy systems due to their selectivity, biocompatibility, and higher transformation efficiency [4], purified proteins, especially enzymes isolated from extremophiles for biocatalytic processes, may have stability issues outside of the natural cellular environments [19]. Certain limitations to large-scale protein production from *Halobacterium* may include growth in salt concentrations above 25% to produce extracellular protease [20], long cultivation times [21], and being evolutionarily adapted to primarily produce acidic proteins to counteract the high saline environments they thrive in [22]. Current evidence shows that *H. salinarum* recover from low salt stress and multiply once high salt concentrations are restored, indicating that their proteome can counter with compensatory mechanisms to withstand fluctuating environmental conditions [23]. Thus, to truly harvest the bioelectrical energy from complex multienzymatic reactions within organisms such as *H. salinarum,* using a whole organism-MFC would be best over biomolecule-driven fuel cells, especially for *in situ* applications.

Recent interest in the power generation of single species halophiles over mixed communities has also taken hold [1, 9]. For example, Shi et al. used Luria Bertani broth at 1% and 4% NaCl concentrations to test the bioelectric output of *Anditalea andensis* an alkaliphilic halotolerant bacterium and achieved a total current capacity of 0.5 microamp/cm^2^ over 120 hours [24]. As per mathematical modelling of *Halobacterium's* respiratory pathway using differential equations, researchers predicted that the organism would be able to generate 280 mV [25]. Furthermore, halophile microbial electric systems can be used to remediate and/or monitor environments for pollutants. *Halobacterium* was shown to degrade crude oil and pure hydrocarbons [26, 27], while recently, a disposable cathode-attached MFC system, with 1.71 M salt and an anodic species *Salinivibrio* in alginate capsules, showed higher sensitivity in monitoring chemical oxygen demand compared to an air-breathing cathode MFC [28]. Proteomic studies also reveal that *H. salinarum* can readjust its biofilm architecture in the presence of metal ions and that nickel treatment increased the organism's biofilm [29]. Collectively, these findings speak to not only the versatility of electrogenic halophiles, but also their potential importance in biomonitoring and bioremediation of polluted hypersaline environments [30].

Although the bioelectric properties of archaea, including halophiles, have been studied, as previously mentioned, no study, to the best of our knowledge, has determined the performance of an *H. salinarum* MFC system. We utilized *H. salinarum* NRC-1 as our anodic organism, as it has well characterized growth and genetic and biochemical properties [14, 31, 32]. MFCs are traditionally conducted under nonoscillating conditions; however, oscillation or agitation is important to increasing microbial growth and is routinely used in growing *H. salinarum* [31, 33]. We hypothesized that the organism should be electrogenic under both oscillating and nonoscillating conditions, as, previously, agitation has been shown to increase biofilm growth of certain wastewater isolates on glass [34] and on carbon cloth electrode surfaces relative to static conditions [35]. The latter study also noted a loss in biofilm reduced power generation in MFC systems [35]. In addition, we tested whether power generation by *H. salinarum* was affected in a closed microbial system under hypersaline conditions over a longer period. Our findings show sustained voltage production in a closed microbial system for up to 6 weeks, highlighting the potential for self-sustaining power generation upon scaling with *H. salinarum* as an anodic organism.

## 2. Experiment

### 2.1. Preparation of Halobacterium Broth

*H. salinarum* 19700 was grown in *Halobacterium* broth (#213) as per ATCC recommendation. 1 L of broth was prepared by mixing 500 mL of a salt mixture into 500 mL of yeast/tryptone mixture. The salt mixture contained 250 g NaCl, 10 g MgSO_4_.7H_2_O, 5 g KCl, 0.2 g Cacl_2_.6H_2_O (Sigma Aldrich) added to 500 mL of distilled water. Separately, 10 g of yeast extract (Fisher Scientific) and 2.5 g tryptone (Becton Dickinson) were added to 500 mL of distilled water, and the pH was adjusted, as required. Both solutions were aseptically mixed together after autoclaving. *Halobacterium* broth was further adjusted postautoclaving, to pH 5.4, 6.4, 7.4, 8.4, and 9.4. 1 M HCl and 1 M NaOH were used to acidify and alkalize the media, respectively.

### 2.2. Determining the Optical Density and Cell-Free Final pH of *H. salinarum*

*H. salinarum* was grown in *Halobacterium* broth at different pH at an inoculum ratio of 1 : 49 in 125 mL flasks. The inoculated broths were incubated in an orbital shaker (MaxQ 4000 Thermo Fisher) set at 100 rpm for 7 days at 37°C. Optical density readings at 590 nm were taken on day 3 and 7 using a Spectronic 20D+. Final pH readings were evaluated to indirectly measure metabolic activity of the organism grown under different pH. Cell free extracts were prepared from the 7^th^ day culture for final pH measures. 5 mL of culture was centrifuged (Baxter 2630 Megafuge), at 1500 RCF for 10 minutes, and the supernatant was separated from the pellet and filtered using a 0.45 *μ*m membrane filter to obtain cell free extracts.

### 2.3. Polarization Curve

The polarization curve data was collected by measuring the voltage drop across various external resistors to the MFC under nonoscillating conditions. A set of resistors with a range of 220 Ω to 1 MΩ was selected as an external resistor. The Track-It™ DC Voltage and Current Data Logger (Monarch Instrument) was used to measure the voltage drop across the external resistor after the stability of the voltage was ensured. The current was calculated using Ohm's law and the internal resistance and, accordingly, internal loss of the MFC estimated from the slopes of the V-I curve across different regions of the polarization curve. The power density curve was plotted by using the measured voltage and calculated current normalized to the anode surface area. The polarization curve was performed on day 6 of normal operation.

### 2.4. Dual-Chambered MFC Set Up with *H. salinarum* under Oscillating and Nonoscillating Conditions

An H-shape dual-chambered MFC, without an electron acceptor at the cathode, was constructed using two 100 mL Pyrex-glass bottles (Adams & Chittenden Scientific Glass) connected by a tube containing a clamped Nafion™ XL proton exchange membrane (PEM) with a surface area of 4.91 cm^2^ and thickness of 0.0275 mm (Fuel Cell). Anode and Cathode were 4 mg/cm^2^ platinum black carbon cloth with surface area of 5 or 10 cm^2^, and a thickness of 0.41 mm with the electrical resistivity of <33 mΩcm^2^ (Fuel cell). The *H. salinarum* broth at pH 6.4 or 7.4 was used as both anolyte and catholyte. Track-It™ DC Voltage and Current Data Logger was used for data measurements. Anode and cathode of the dual-chambered MFC were connected through a 100 kΩ resistor externally. Data on voltage measurements across the 100 kΩ were used for power density (mW/m^2^) calculation. The power density is defined as

(1) $$\begin{array}{c} \text{Power density}\left(\frac{mW}{m^{2}}\right)=\frac{I\times V}{A}, \end{array}$$

where *I* is the current in (mA), *V* is the voltage in (volts), and *A* is the effective surface area of the electrode in (m^2^) [36]. Power density was normalized to the anode effective surface area [37].

### 2.5. Stacked MFC Series under Oscillation

To increase the output voltage, a series stacked MFC configuration was also designed and tested by using two identical sets of dual-chambered MFCs with 10 cm^2^ electrodes. The anode of the first dual-chambered MFC was connected to the cathode of the second dual-chambered MFC. The cathode of the first dual-chambered MFC and the anode of the second dual-chambered MFC were connected through the external resistor. The Track-It™ DC Voltage and Current Logger was connected across the external resistor to measure the output voltage of the stacked series dual-chambered MFCs over fourteen days under oscillation.

### 2.6. Statistical Analysis

Data was analyzed for statistical significance using GraphPad Prism 9.0 software. The effect of media pH on growth parameters was analyzed using a nonparametric ANOVA Kruskal-Wallis test, with Dunn's multiple comparison. Differences in voltage output due to media pH, electrode size, and oscillation were tested using an unpaired *T*-test with Welch correction.

## 3. Results

### 3.1. Effect of pH on *H. salinarum* Growth

OD readings were established to control the time of transfer of organism to the anode chamber. Growth after 3 days of incubation was determined to be the best for MFC inoculation as *H. salinarum* achieved an approximate OD of 1, regardless of initial media pH (supplementary Table 1), and based on pigment development (supplementary Figure 1). OD readings after 3 or 7 days of incubation from media with different pH showed no significant difference based on the Kruskal-Wallis analysis. Changes in media pH after 7 days of incubation, from cell free extracts, were also analyzed to indirectly evaluate metabolic activity and found to be statistically insignificant. However, the final pH readings did rise on average by 2 units, indicating a shift to alkaline conditions (supplementary Table 1). Growth of *H. salinarum* in pH 5.4 and 9.4 was comparatively very slow, with OD readings peaking after 7 days of incubation to 1.20 and 0.05, respectively. The poor growth of the organism, coupled with fluctuations in media pH, once adjusted to 5.4 or 9.4, led us to discontinue evaluating growth and subsequent MFC output at these pH levels.

### 3.2. Polarization Curve Data

**Figure 1:**
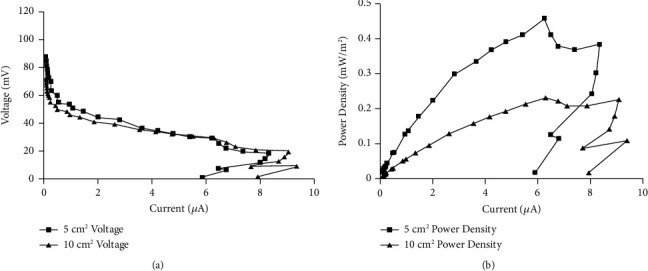
(a) shows the polarization curve, while (b) shows the corresponding power curves for MFCs with 5 cm^2^ and 10 cm^2^ electrode surface area measured on day 6 of normal operation from a dual-chambered MFC at pH 6.4, with *H. salinarum* as the anodic organism at a ratio of 1 : 24, maintained at 37°C under nonoscillating conditions.

Figure 1(a) shows the polarization curves of two dual-chambered MFCs with electrode size of 5 cm^2^ and 10 cm^2^ under nonoscillating conditions with the anolyte and catholyte media at pH 6.4. The vertical axis shows the output voltage of the MFC in mV, the horizontal axis, and the current of the MFC in *μ*A. Figure 1(a) demonstrated a rapid drop accompanied by a loss of 20 mV to 25 mV in the activation region, a relatively slower drop but with a larger loss of 40 mV to 50 mV in the Ohmic region, and a fast drop with a loss of around 15 mV to 20 mV at the mass activation region. The total internal resistance was estimated by measuring the slops of V-I curves at 5000 Ω and 3700 Ω for 5 cm^2^ and 10 cm^2^ electrode surface area, respectively. Total internal resistance is heavily influenced by Ohmic loss, which is the most important loss in MFCs. Figure 1(a) also shows a slightly higher total internal resistance for MFC with a 5 cm^2^ electrode surface area compared with a 10 cm^2^.  Figure 1(b) shows the corresponding power curves with the vertical axis as the output power density (mW/m^2^) and the horizontal axis as the current (*μ*A) of the MFCs. The curves showed a close symmetrical behavior of power density to current, and a maximum value of 0.459 mW/m^2^ and 0.233 mW/m^2^was achieved for 5 cm^2^ and 10 cm^2^ electrode surface area, respectively. 5000 Ω and 3700 Ω for 5 cm^2^ and 10 cm^2^ electrode surface area, respectively, as an external resistance value can transfer the maximum power of MFC into the load resistance. However, we chose a 100 kΩ to establish power generation as an open circuit MFC in order to establish maximum voltage output for future addition of a booster, which would have a very high input impedance. Given that the voltage produced by MFCs is usually too low for device operations, using a boost converter can help in making the MFC, under open circuit voltage, operational [38].

### 3.3. Anolyte and Catholyte pH Affect MFC Voltage Output over a Longer Run in a Closed MFC System

**Figure 2:**
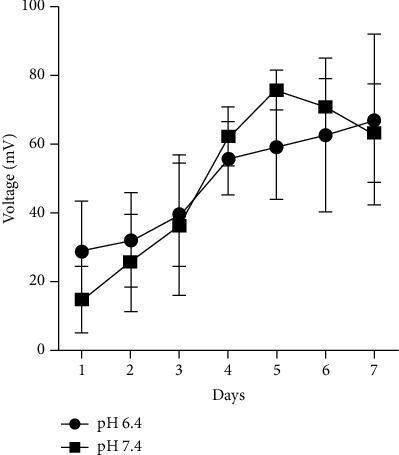
Voltage (mV) achieved over 7 days from two-chambered MFC units at different pH (6.4 and 7.4), with *H. salinarum* as the anodic organism at a ratio of 1 : 24, maintained at 37°C, with constant shaking at 50 rpm. Each MFC unit consisted of 5 cm^2^ electrodes. The data points represent the daily average voltage (mV ± SEM) of readings recorded every 30 minutes for 7 days from 3-4 separate experiments. There was no significant difference (*p*=0.9663) in voltage due to pH based on an unpaired *T*-test with Welch correction.

**Figure 3:**
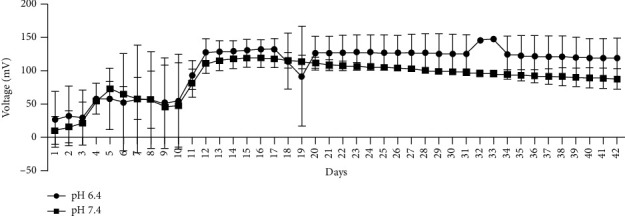
Daily average voltage (mV) recordings from a dual-chambered MFC (5 cm^2^ electrodes) unit with *H. salinarum* as the anodic organism at either pH 6.4 or 7.4 at a ratio of 1 : 24, maintained at 37°C, with constant shaking at 50 rpm. Each data point is the daily average voltage (mV) recorded every 30 minutes over 42 days from two separate trials at each pH. The voltage was higher (*p*=0.01) when the anolyte and catholyte were at pH 6.4 compared to 7.4, as per unpaired *T*-test with Welch correction.

The effect of pH of the anolyte and catholyte on voltage output by *H. salinarum* was evaluated. All MFCs were tested as a closed MFC system, where the system did not receive fresh media or removal of waste periodically, as done traditionally. As shown in Figure 2, after 7 days, MFCs at pH 6.4 and 7.4 showed no difference (*p*=0.9663), achieving a maximum voltage of 67.34 ± 25.03 and 63.45 ± 14.34 mV, respectively. However, when the MFC was run over 42 days, steady voltage readings were obtained under a closed system. The overall voltage was significantly (*p*=0.01) lower at pH 7.4 compared to pH 6.4 (Figure 3). The average maximum power density at pH 6.4 was higher at 0.4356 mW/m^2^ (Table 1) relative to pH 7.4 (0.3559 mW/m^2^) over the 42-day run. The sudden drop and peak in readings, as seen in Figure 3, were taken as a possible distortion and not considered in the calculations. In contrast, the MFC with anolyte and catholyte media at pH 8.4 could only reach a maximum voltage of 13.07 mV over 7 days (data not shown). Based on these results, further optimization of dual-chambered closed MFCs was run using anolyte and catholyte media at pH 6.4.

### 3.4. Larger Electrode Size Affects MFC Performance under Oscillating Conditions

**Figure 4:**
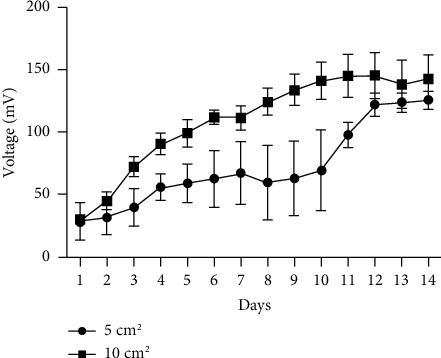
Comparative effect of the surface area of different size platinum carbon cloth electrodes on voltage (mV ± SD) output by *H. salinarum* at pH 6.4 over a period of 14 days at 37°C with oscillation. Each data point is the daily average voltage (mV) recorded every 30 minutes from 2–4 separate experiments. An unpaired *T*-test with welch correction determined doubling the electrode size has significant (*p*=0.01) effect on voltage under oscillating conditions.

**Figure 5:**
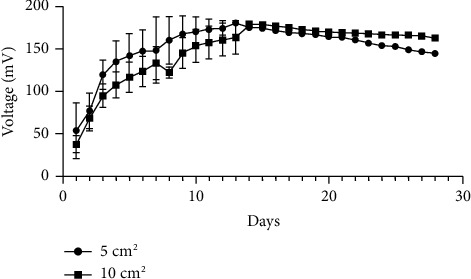
The effect of the surface area on voltage (mV ± SD) output by *H. salinarum* over 28 days at 37°C without oscillation. Each data point is the daily average voltage (mV) recorded every 30 minutes from 1–4 separate experiments. Electrode surface area had no effect (*p*=0.5944) on voltage under nonoscillating conditions based on an unpaired *T*-test with Welch correction.

**Figure 6:**
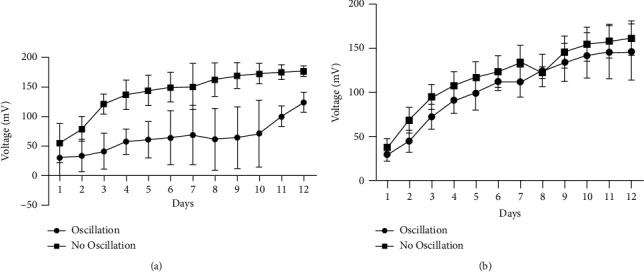
The effect of oscillating vs nonoscillating conditions on MFC voltage (mV ± SD) output by *H. salinarum* at 37°C using either (a) 5 cm^2^ or (b) 10 cm^2^size platinum carbon cloth electrodes. Nonoscillating conditions significantly (*p* < 0.0001) affected voltage with a 5 cm^2^ electrode size, while there was no change (*p*=0.3709) with 10 cm^2^ sized electrodes as per an unpaired *T*-test with welch correction.

The amount of power that is generated in an MFC system is affected by the surface area of the cathode relative to that of the anode and the surface of the membrane. The power produced in this system was compared based on similarly sized anodes, cathodes, and membranes in our experiment. Figures 4 and 5 show the effect of using 5 cm^2^ versus 10 cm^2^ electrodes under either oscillating or nonoscillating conditions, respectively. An unpaired *T*-test with Welch correction showed that doubling the electrode size had a significant (*p*=0.01) effect on voltage under oscillating conditions over 14 days (Figure 4), while voltage output was not affected by electrode size under nonoscillating conditions (*p*=0.5944) (Figure 5). However, the electrode size of 5 cm^2^ produced higher (*p* < 0.0001) voltage under nonoscillating compared to oscillating conditions (Figure 6(a)), but the effect of motion is mitigated when the electrode size is doubled (Figure 6(b)).

### 3.5. Higher Voltage Production in Stacked MFC with Larger Electrode Surface Area

**Figure 7:**
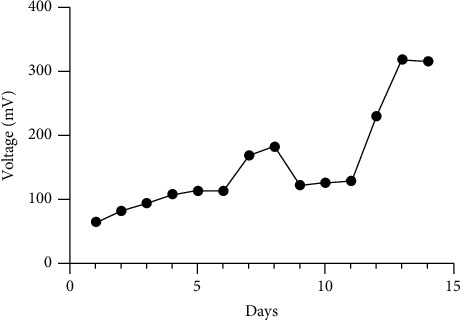
Voltage output from a double-stacked in series MFC unit containing an electrode size of 10 cm^2^ with *H. salinarum* at pH 6.4 over 14 days of incubation at 37°C at 50 rpm oscillation. Each data point is the daily average voltage (mV) recorded every 30 minutes over 14 days.

The H-shape dual-chambered MFC systems are acceptable for basic parameter research, but they typically produce low power densities. As such, a series or parallel connection of multiple MFCs can increase the overall power [39]. We chose to test a series configuration from stacked MFCs, under oscillation, as the output voltage in series approximates the addition of individual MFC voltages, while the current of a series stacked MFC remains the same as that of a single-mode MFC [40–42]. The stacked series generated a higher voltage across the load resistor at 318.88 mV (Figure 7) almost twice compared to an individual dual-chambered MFC (145.7 ± 18.3 mV) (Table 1). In addition, power density was approximately 6X higher (1.2672 mW/m^2^) compared to an individual dual-chambered MFC (0.274 ± 0.07 mW/m^2^) (Table 1).

### 3.6. Power Density Based on Electrode Size and Nonoscillation

Table 1 shows the maximum power density calculated based on maximum voltage and its corresponding current based on electrode surface area, and oscillating or nonoscillating conditions at 37°C. The surface area of the identical anode and cathode electrodes at 5 cm^2^ and 10 cm^2^, with an effective surface area of 4 and 8 cm^2^, respectively, was used in power density calculation. By using a 100 kΩ load resistor, the voltage, current, and power density behaved the same across the experiments. The overall difference in MFC performance due to nonoscillation compared to oscillation doubled the power densities in similar electrode sized MFCs over a shorter run. The best single-mode performance was observed over 28 days under nonoscillating conditions and electrodes of 5 cm^2^ with a maximum power density of 0.8184 mW/m^2^. However, the highest maximum power density was obtained over 14 days with oscillation at 1.2672 mW/m^2^, which was the result of two dual-chambered stacked series MFCs with 10 cm^2^ electrodes.

## 4. Discussion

MFC performance is tied to physical properties of the unit including structure, component material, and anodic bacterial community, as well as environmental factors such as pH, salinity, and temperature. These factors in turn can affect the internal resistance of the system [43] and subsequently MFC output. Our aim was to understand the efficacy of an MFC system with *H. salinarum* NRC-1 grown in a closed-microbial system under oscillating and nonoscillating conditions. We obtained the highest maximum voltage (318.8 mV) and power density (1.2672 mw/m^2^) with two dual-chambered stacked series MFCs, with 10 cm^2^ electrodes, over 14 days under oscillation. We did not compare the performance of the stacked series under nonoscillating conditions, which produced the best MFC performance in single-mode operation. Nonoscillating conditions are the norm in MFCs, with operation being set up to either receive sludge [44], batch-fed with nutrients [45] or sediments [8]. However, these processes can introduce periodic disturbances at the electrode surface. Examining MFC output under oscillation better represented real-world usage, and in addition, oscillation helped aid the growth and pigment production of *H. salinarum*, as high salinity reduces dissolved oxygen in the media. Although nonaerated systems compared to aerated systems favor the abundance of electrogenic bacteria on anodic surfaces [46], we noted that the electrode surface was well coated after 14 days of oscillation (supplementary Figure S2). While the current study was not equipped to analyze anodic biofilm composition, our data supports our initial thinking that *H. salinarum* is capable of sustained voltage production under both oscillating and nonoscillating conditions, as an anodic organism. Future studies, to help scale up output under oscillation, should evaluate changes to the surface area to volume ratio, inoculum concentration, and increasing the number of units in the stacked series.

We had anticipated a difference in voltage due to pH, but trying to maintain media pH below 6.4, and above 8.4 proved difficult. Even though pH had no effect on growth, the MFC readings at pH 8.4 were lower compared to those observed at pH 6.4 and 7.4. Although not enough data is present for us to make assumptions, our finding suggests that higher pH may lower voltage output over a longer time under hypersaline conditions. Further experimentation is required to understand how high pH might affect MFC function in a closed microbial system at high salt concentrations. Anodic oxidation reactions are strongly acidifying, which is why alkaline feeds such as acetate [5, 47] and urine [48, 49] are favored in microbial electrical systems, but the results on the effect of pH on MFC performance are contradictory. An earlier study showed the highest current to be produced at pH 7 [50]; similarly, voltage output decreased in an MFC with a switchable power release, used to control combinations of pH and temperature, when the pH of the system was lowered from 7 to 5 [51]. In contrast, Pham et al. demonstrated changing the pH to either 5.5 or 8.8 of an aquaculture pond water, integrated to a halophilic sediment bioelectrical system, increased electrical conductivity by 1.5-fold [52]. Alternatively, *Halobacterium* media, used in our study as the anolyte and catholyte, is not a buffering solution as it does not possess any salts that act as a weak acid/conjugate base; however, recent data on the relationship between high molar salt solutions and increased pH has led to the hypothesis that NaCl at concentrations above 1.8 M acts basic, which in turn could affect the pH of the salt solution [53], suggesting that pH effects under hypersaline conditions are complicated and require more understanding.

## 5. Conclusion

Our findings provide preliminary understanding to the bioelectric functionality of an *H. salinarum* MFC system. Although the power density produced in H-shaped dual-chambered MFCs, as employed in our study, is typically limited by high internal resistance and electrode-based losses, optimization can be realized using a single chamber MFC, which can reduce pH decreases at the anode [54] and allow for biosensor application. In addition, modifications to carbon-based electrodes can help enhance anode-biofilm interaction, increase conductivity, and improve MFC performance [55]. Early results from our lab have shown *H. salinarum* to potentially degrade 10 ppm poly-chlorinated biphenyls (Arclor 1242) (data not shown). Although further analysis is required to fully understand the biodegradative capability by *H. salinarum*, a potential *Halobacterium* MFC-biosensor holds promise in environmental cleanup in marine environments.

## Figures and Tables

**Table 1:** Summary of results on the effect of electrode surface area and oscillation on voltage, current, and power density generation.

Total number of days	Oscillation/No-oscillation	Volume (ml)	pH	Effective surface area of electrode (m^2^)	Area of membrane (m^2^)	Max Voltage (mV)	Current at max power density (*μ*A)	Max power Density (mW/m^2^)
7	Oscillation	2 × 100	6.4	0.0004	0.00049	67.3 ± 25.0	0.7 ± 0.3	0.160 ± 0.07
14	Oscillation	2 × 100	6.4	0.0004	0.00049	127.1 ± 7.2	1.0 ± 0.072	0.410 ± 0.05
42	Oscillation	2 × 100	6.4	0.0004	0.00049	132.0	1.3	0.436
14 14	Oscillation Oscillation	2 × 100 4 × 100	6.4 6.4	0.0008 0.0008	0.00049 0.00049	145.7 ± 18.3 318.80	1.0 ± 0.1 3.2	0.274 ± 0.07 1.267^*∗*^
28	No-oscillation	2 × 100	6.4	0.0004	0.00049	180.9	2.0	0.8184
28	No-oscillation	2 × 100	6.4	0.0008	0.00049	179.2	2.0	0.4014

^*∗*^Max power density was calculated from the two dual-chambered stacked series MFC units containing an electrode size of 10 cm^2^.

## Data Availability

The data associated with this research are available from the corresponding author upon request.

## Supplementary Materials

Supplementary Materials Supplementary Table 1. Optical Density of *H. salinarum* grown in Halobacterium broth for 7 days at different pH at 37°C with shaking (100 rpm). Supplementary Figure 1. Effect of media pH on growth of *H. salinarum* over 7 days of incubation at 37°C with shaking (100 rpm). Top panel images represent growth observed after 3 days of incubation, while the bottom panel shows growth on the 7^th^ day of incubation. Image pairs A-B, C-D and E-F show growth in pH 6.4, 7.4 and 8.4, respectively. Supplementary Figure 2. Panel A is an image of the dual-chambered MFC set up with Halobacterium media at pH 6.4 on either side and growth of *H. salinarum* on the anodic side. Panel B shows the 10 cm^2^ anodic electrode surface from the MFC on the left, following 14 days of incubation at 37°C at 50 rpm oscillation.
