# Supplementary material for: Halobacterium salinarum NRC-1 Sustains Voltage Production in a Dual-Chambered Closed Microbial Fuel Cell
Source: ScientificWorldJournal. 2022 Sep 12;2022:3885745. doi: 10.1155/2022/3885745 (PMC9484973; doi:10.1155/2022/3885745)
Supplement: Supplementary Materials — Supplementary Table 1. Optical Density of H. salinarum grown in Halobacterium broth for 7 days at different pH at 37°C with shaking (100 rpm). Supplementary Figure 1. Effect of media pH on growth of H. salinarum over 7 days of incubation at 37°C with shaking (100 rpm). Top panel images represent growth observed after 3 days of incubation, while the bottom panel shows growth on the 7th day of incubation. Image pairs A-B, C-D and E-F show growth in pH 6.4, 7.4 and 8.4, respectively. Supplementary Figure 2. Panel A is an image of the dual-chambered MFC set up with Halobacterium media at pH 6.4 on either side and growth of H. salinarum on the anodic side. Panel B shows the 10 cm2 anodic electrode surface from the MFC on the left, following 14 days of incubation at 37°C at 50 rpm oscillation. [file 3885745.f1.docx]

**Supplementary Table**

**Suppl. Table 1**: Optical Density of ***H. salinarum*** grown in Halobacterium broth for 7 days at different pH at 37˚C with shaking (100 rpm)

| Measurement | pH 6.4 | pH 7.4^*^ | pH 8.4 | Significance |
| --- | --- | --- | --- | --- |
| OD after 3 days^1^  OD after 7 days^2^  Final pH^3^ | 1.0±0.4  1.5±0.1  8.2±0.2 | 0.8±0.2  1.3±0.2  8.4±0.1 | 0.8±0.2  1.4±0.04  7.9±0.4 | p=0.8952  p=0.6329  p=0.4333 |

Each measure^1,2,3^ is shown as the average ± SEM from three separate experiments. ^*^Growth measures from pH 7.4 are an average of four separate experiments. The effect of initial media pH on OD and final pH was not significant as determined by the Kruskal-Wallis test.

**Supplementary Figures**


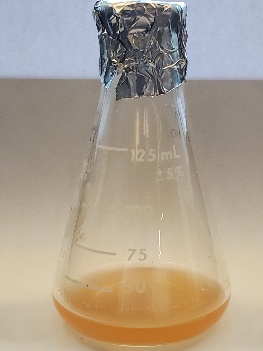

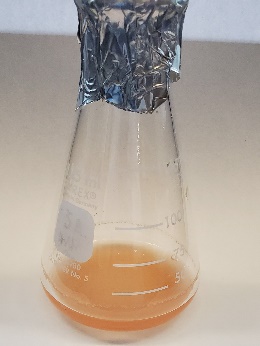

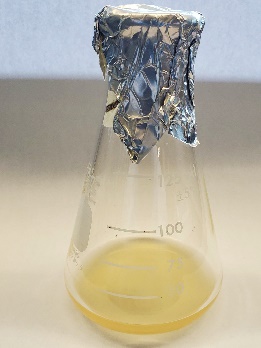

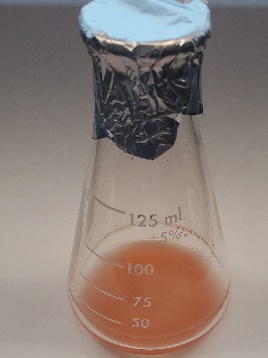

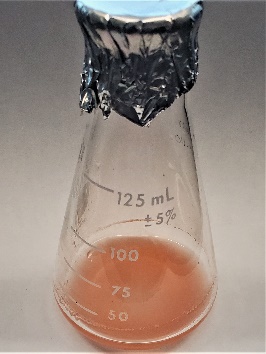

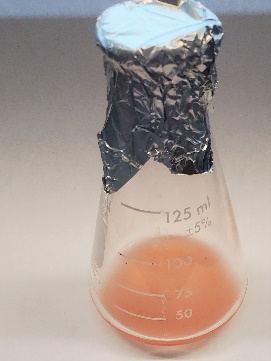


D

F

B

E

C

A

**Suppl. Figure 1**. Effect of media pH on growth of *H. salinarum* over 7 days of incubation at 37˚C with shaking (100 rpm). Top panel images represent growth observed after 3 days of incubation, while the bottom panel shows growth on the 7^th^ day of incubation. Image pairs A-B, C-D and E-F show growth in pH 6.4, 7.4 and 8.4, respectively.


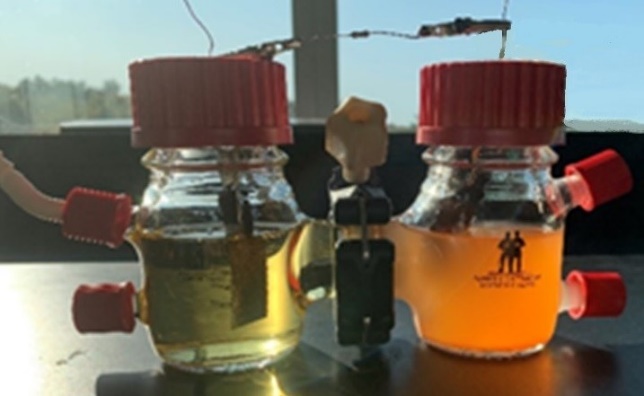

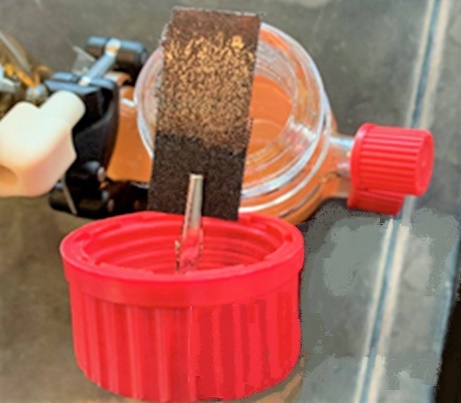


B

A

**Suppl. Figure 2**. Panel A is an image of the dual-chambered MFC set up with Halobacterium media at pH 6.4 on either side and growth of *H. salinarum* on the anodic side. Panel B shows the 2x5 cm^2^ anodic electrode surface from the MFC on the left, following 14 days of incubation at 37˚C at 50 rpm oscillation.
